# Lessons learned from implementing the pilot Micronutrient Powder Initiative in four districts in Ghana

**DOI:** 10.1186/s40795-020-00382-3

**Published:** 2020-11-09

**Authors:** Frank Kyei-Arthur, Ruth Situma, Jevaise Aballo, Abraham B. Mahama, Lilian Selenje, Esi Amoaful, Seth Adu-Afarwuah

**Affiliations:** 1 Regional Institute for Population Studies, University of Ghana, Legon, Accra, Ghana; 2 UNICEF Ghana, 4-8th Rangoon Close, Cantonments, Accra-North, Ghana; 3 Ghana Health Service, Accra, Ghana; 4 Department of Nutrition and Food Science, University of Ghana, Legon, Accra, Ghana

**Keywords:** Anemia, Micronutrient deficiencies, Micronutrient powder, Micronutrient powder initiative, Lessons learned

## Abstract

**Background:**

Micronutrient deficiencies affect many children in low-income settings due primarily to over-reliance on complementary foods low in nutrients. Home-fortification (HF) could improve children’s diet quality in these settings. The Ghana Health Service, supported by UNICEF, integrated the pilot Micronutrient Powder Initiative (MPI) into Child Welfare Clinic (CWC) services in four districts (Tain, Tolon, Talensi, and Ho West), whereby micronutrient powder (MNP) is supplied for HF for children aged 6–23 months attending CWCs. This study’s main aim was to identify the facilitators, barriers and “lessons learned” after 2 years of program implementation.

**Methods:**

This was a qualitative cross-sectional study. MNP distributed and children enrolled were obtained from program records. Primary data were collected from November to December 2019 and included, by district: interviews with senior program staff; key informant interviews and focus group discussion with caregivers in each of 6 sub-districts; and discussions workshop with frontline staff from at least 10 health facilities. Besides field notes, all interactions were audio-recorded and transcribed verbatim. Qualitative data were analyzed using NVivo10.

**Results:**

The MPI remains on-going, with about 2.5 million MNP sachets distributed to nearly 30,000 children within 90 district-months. Caregivers generally accept the MNP; reported positive responses in children include: “increased appetite”, “less frequent illnesses, “increased energy/strength”, “increased weight”, and “walking independently relatively early”. Main facilitators are: generally regular MNP availability; increased patronage of CWC services; various contact points for supplying MNPs; fairly strong social mobilization strategy; good integration of MPI with CWC services; “one-on-one” counselling for caregivers reporting side effects; and tracing caregivers to address concerns and monitor adherence. Main barriers are: lack of counselling materials; caregivers’ suspicions towards the program; absence of refresher training for frontline workers; and perceived MNP side-effects. Key lessons learned are: incorporating MNPs into CWC services is feasible, acceptable, and could reduce child micronutrient deficiencies in program districts; and MPI’s success requires stronger community sensitization, equipping frontline workers to advise caregivers and manage side-effects, and consciously identifying and managing logistical challenges.

**Conclusion:**

Further research is needed to evaluate the effectiveness of the MPI in reducing micronutrient deficiencies among 6–23-months-olds in Ghana.

## Background

Anemia and micronutrient deficiencies in children are major public health problems in many low-and middle-income countries [1]. In 2017, an estimated 36% of children 6–59 months of age in Ghana were anemic, and about 22% had iron deficiency [2]; higher anemia prevalence of 66% among children 6–59 months of age in Ghana was reported in 2014 [3]. Anemic and/or micronutrient-deficient children may suffer from poor growth, impaired cognitive development, and increased risk of morbidity and mortality [4].

One reason for the high prevalence of anemia and micronutrient deficiency in children in Ghana is the dependence on complementary foods based on cereal paps low in nutrients [5]. In such setting, home (point-of-use) fortification of complementary foods might be a pragmatic strategy for improving diet quality, and thereby treat and/or prevent anemia and micronutrient deficiencies in this vulnerable group. Home fortification has several advantages: it can be targeted to specific groups or households; be incorporated into the usual complementary diet without necessitating a major dietary modification [6]; and be used to deliver micronutrients to vulnerable groups in remote settings where fortified products are not affordable or available.

In late 2017, the Ghana Health Service (GHS), with the support of UNICEF, began implementing the Micronutrient Powder Initiative (MPI) in 4 districts across 4 regions of the country. This pilot program uses various routine health service contacts, particularly the monthly Child Welfare Clinics (CWCs) or growth monitoring and promotion sessions (GMPs), to supply mothers and caregivers of children 6–23 months of age with micronutrient powders (MNPs) for home (point-of-use) fortification of complementary foods for those children. This intervention is consistent with international efforts at combating micronutrient deficiencies [7] and improving child nutrition, health, development and survival [8–10].

The use of MNPs to enrich the home-prepared foods for infants and young children is not new in Ghana. In 2001, MNPs containing iron and vitamin C were shown to be efficacious in the treatment of anemia in the Kintampo area [11]. Since then, the efficacy of MNPs have been investigated or demonstrated in several other randomized trials in Ghana [12–15]. For reducing anemia and micronutrient deficiencies in young children, MNPs have been distributed in the context of pilot interventions in many countries [16, 17] such as Ethiopia [18], Mali [19], Peru [20], Rwanda [21], and Uganda [21].

In Ghana, complementary feeding counselling has been part of CWC sessions for decades, but the MPI is the first time the GHS has combined routine complementary feeding counselling with the use of MNPs delivered through routine health services in an attempt to improve micronutrient intakes and address anemia in children on a relatively large scale. While the program remains ongoing 2 years since its inception, the facilitators and barriers to its implementation have not been identified, neither have the lessons learned been documented. The inability to document program lessons learned in nutrition is often a major gap in developing countries. The objective of this study was to examine the MPI in the 4 districts in Ghana, with a focus to (a) describe the experiences of beneficiaries and implementers of the program, (b) document successful strategies used by the program implementers, (c) identify the facilitators and barriers to the program implementation, and (d) discuss the lessons learned.

### Theoretical framework

The theoretical framework guiding this study was the conceptual framework described by Siekmans et al. for the analysis of how MNPs could strengthen complementary feeding practices [22]. According to this framework, MNP’s potential to influence complementary feeding practices may be based on several components, key among which are the delivery of MNPs at the health facility and community levels and actions of mothers and caregivers towards adhering to MNP feeding recommendations at the household level. In this study, our intent was to investigate the implementation of the MPI at these two levels.

## Methods

### The micronutrient powder initiative (MPI)

The ongoing pilot Micronutrient Powder Initiative (MPI) is being implemented in the Tain district (Brong-Ahafo region at the time the program began), Tolon district (Northern region) Talensi district (Upper East region), and Ho West district (Volta region). The Tain district is close to the Ghana border with *La Côte d’Ivoire;* its capital, Nsawkaw, is about 432 km north-west of Accra, the national capital. The capital of the Tolon district, Tolon, and that of the Talensi district, Tongo, are about 647 km and 800 km, respectively, north of Accra, while the Ho West district shares borders with the Republic of Togo, and its capital, Dzolo-Kpuita, is 175 km north-east of Accra. The total populations of the 4 districts range from about 73,000 in Tolon to 95,000 in Ho West (http://www.ghanadistricts.com/). In 2014, the anemia rates among children < 5 years in the regions in which the districts are located were estimated at 63% for Tain, 82% for Tolon, 74% for Talensi, and 70% for Ho West [3].

The MNP being used in the MPI contains 15 micronutrients per 1 g sachet, including Vitamins A (400 μg RE), D3 (5 μg), E (5 mg TE), B1 (0.5 mg), B2 (0.5 mg), B3 (6 mg), B6 (0.5 mg), folic acid (90 μg), B12 (0.9 μg), C (30 mg), iron (10 mg), zinc (4.1 mg), selenium (17 μg), copper (0.56 mg) and iodine (90 μg) (DSM Nutritional Products Europe Ltd., CH – 4002 Basel). This composition is based on the World Health Organization (WHO) guidelines [7] providing generally one FAO/WHO Recommended Nutrient Intake, RNI (i.e. the daily recommended nutrient intake) for children 1–3 years [23]. Iron is present as encapsulated ferrous fumarate [7, 24]. According to the product details at the UNICEF Supply Catalogue website (https://supply.unicef.org/s0000225.html), the MNP is odorless and off-white or slightly yellow, and has a bland taste so that it has a minimal impact on the taste, smell or color when mixed with food.

As part of each district’s routine medical supplies, the MNPs are typically “picked-up” from the Regional Medical Stores, RMS (serving as the collection point) by the District Nutrition Officer (DNO) and deposited at the District Health Directorate (DHD) store, from where staff dispatched from the various health facilities (polyclinic, clinics, CHPS compound, etc.) collect the MNP. In some cases, the RMSs deliver the MNP to districts and health facilities via “scheduled delivery”, but this happens less often. From each health facility, Community Health Nurses distribute MNP to mothers and caregivers at various contact points, for home (point-of-use) fortification of complementary foods for children 6–23 months of age. The main point for MNP distribution is the CWCs or GMPs held by the health facilities at various permanent (or “static”) and mobile (or “outreach”) locations, which children 0–59 months of age attend monthly. Another contact point for MNP distribution is the out-patient departments of the various health facilities.

The MNP distribution is done alongside the routine provision of complementary feeding counselling to all mothers and caregivers attending the CWC or GMP. As is written as well as illustrated pictorially on the sachet packets, mothers and caregivers receiving the MNPs for their 6–23 mo-old children are advised to give the child 1 sachet per day by mixing “the content of one sachet into a portion of solid or semi-solid food before serving”. There is, however, no description or warning of side effects on the sachet packets, and therefore, it is conceivable that the Community Health Nurses delivering the MNPs would typically not warn mothers/caregivers of any possible side effects. Currently, there are no teaching aids or behavior change materials specifically developed for MNP distribution and consumption. Mothers are introduced to MNPs through infant and young child feeding (IYCF) counselling given using the Ghana Ministry of Health IYCF counseling cards [25]. These counselling cards encourage the commencement of complementary feeding of infants at 6 mo of age; provision of a variety of foods that are easy for infants to eat or swallow; assisting infants to eat complementary foods through coaxing but not forced feeding; and ensuring hygiene [25].

Within the communities, MNP consumption is monitored by Community Health Nurses and Community Health Workers (trained low level cadre of health workers recruited from the communities where they live) [26] through routine home visits for delivering basic preventive and curative services at the household level, under the Community-based Health Planning and Services (CHPS) system [26, 27]. A child may stay in the MPI program for a maximum of 18 months. In the GHS’ approach, each child receives 30 sachets of MNP each month for a period of 3 months, followed by 3 months of not receiving any MNPs, and the cycle is then repeated until the child is 24 months of age. According to available guidelines [28], the target is to give 90 sachets of MNPs generally containing one RNI for each micronutrient over a period of 6 months, but the exact frequency of distribution (eg. all 90 sachets at once every 6 months or 30 sachets every other month, etc.) depends on programmatic feasibility [28].

Through the health facilities, the GHS uses such activities as local radio discussions, meetings with local chiefs and opinion leaders, and community durbars to sensitize communities to the MPI. UNICEF supplies the MNPs to the GHS, as well as provides various logistical and operational support capabilities, such as the training of GHS staff involved in the program implementation, and the supply of registers to track MNP acquisition and distribution.

### Lessons learned study design and data collection

This was a qualitative cross-sectional study. During a 2-month period from November to December 2019, data were collected from each of the 4 districts as follows: secondary data on the quantity of MNP sachets received and number of children 6–23 months of age enrolled into the MPI since the beginning of implementation were obtained from the DNO who kept those records as furnished by the health facilities. Next, primary data were collected from 3 sources:

First, interviews were held with at least 5 GHS staff overseeing the MPI implementation, including the DNO (who was responsible for managing the program at the district level) and nurses from the major health facilities distributing the MNPs. Using a semi-structured questionnaire with open-ended questions (Supplementary file 1), information collected included: observations or experiences gained from the program implementation, including challenges or barriers encountered; successful strategies used in the implementation; and lessons learned.

Second, key informant interviews (KIIs) and focus group discussions (FGDs) were conducted with mothers and caregivers who had a previous or current direct experience with feeding MNP to their infants 6–23 months of age. In each of 6 sub-districts within each district, 2 mothers or caregivers serving as key informants were selected to participate in individual KIIs, and up to 10 mothers and caregivers were selected to participate in a FGD. With the help of GHS staff involved with the program implementation in each sub-district, participants were selected for the KIIs or FGDs if they were believed to have considerable knowledge about the MPI and the attitudes of women in the communities towards MNP acceptability and consumption. Semi-structured questionnaires and interview guides prepared for the KIIs (Supplementary file 2) and FGDs (Supplementary file 3) solicited information related to the MPI, including mothers’ and caregivers’ observations or experiences participating in the program and what they had observed about the impact of MNP consumption in their children. It was unknown if any of the KII and FGD participants had purposely stopped feeding MNPs to their children for any reason, given that these participants were from among the list of mothers and caregivers recorded by the CWCs to have previously received, or were currently receiving, MNPs. The GHS staff had no list of those who had refused to try feeding MNPs to their children, and therefore there was none of such participants in the study.

Third, a discussions workshop was conducted in each district, in which at least one frontline staff member from each of 10 health facilities across the district participated. This workshop was used, first, to gather additional information about the MPI not yet captured in the prior interviews with program managers, KIIs and FGDs, and second, to allow the participants to validate, wherever possible, the information already collected through the interviews, KIIs and FGDs. Specific areas discussed at the workshop included those areas covered in the interviews with GHS staff overseeing the program implementation (Supplementary file 1).

In addition to the field notes taken, the interviews and discussions were audio-recorded and later transcribed verbatim. The interviews and workshop discussions with staff of GHS were held in English, whereas the KIIs and FGDs were held in the local languages before being translated into English. Ethics approval for this study was obtained from the Ghana Health Service Ethics Review Committee. Permission to interview the GHS staff was obtained from the Director, Family Health Division, Ghana Health Service.

### Data analysis

The secondary data on quantities of MNPs received and the number of children enrolled into the MPI in each district were summarized using appropriate descriptive statistics. Transcripts and filled questionnaires were cleaned and imported into NVivo10 for analysis (QSR International, Melbourne, Australia). These data were analyzed using thematic analysis approach [29]. All transcripts and open-ended questions in the questionnaire were read in order to understand the data and to identify emerging codes and themes. The organizing themes focused on aspects of the MPI including (a) experiences of beneficiaries (mothers and caregivers) enrolled in the program, (b) experiences of program implementers (staff of GHS), including major challenges to program implementation and successful strategies used, and (c) suggested recommendations from beneficiaries and program implementers. The main experiences, challenges to program implementation, and strategies used were summarized, along with quotes from participants, for clarification and support. In all cases, quotes from multiple data sources (key informants, FGD panelists, and/or health workers) were presented showing triangulation of results, to support the conclusions. The facilitators (factors promoting the implementers’ ability and willingness to execute the program successfully, and mothers’ or caregivers’ ability and willingness to accept and utilize the program as promoted) and barriers (factors militating against successful program implementation or uptake) were also summarized.

## Results

Table 1 shows the summary statistics from the secondary data, along with background details on the primary data collected, by district. At the time of data collection, about 2.5 million sachets of MNP had been received in the 4 districts in a period totaling 90 district-months since the program’s beginning, and nearly 30,000 children 6–23 months of age consisting approximately the same number of males as females had been enrolled to receive the MNP. Ho West received the highest amount of MNPs, followed by Tain district, and then Talensi district. With respect to the number of children enrolled into the MPI, Tain district had the highest number of children, followed by Tolon, while Ho West had the lowest number of children enrolled given, in part, that Tain district was the first and Ho West was the last to start the program. The same number of participants across the 4 districts took part in the KIIs, FGDs and discussion workshops. On average, the number of hours of audio recordings collected per district was more than 9 h for the KIIs, nearly 8 h for the FGDs, and about 2 h for the discussions workshop.

**Table 1:** Summary of secondary data and details of primary data for the 4 MPI districts collected during a 2-month period from November to December 2019

	Tain district	Tolon district	Talensi district	Ho West district
Secondary data on MNP received and distributed^a^				
MPI start-date	Oct 2017	Jan 2018	Dec 2017	Jan 2018
Total MNP sachets received by health facilities	648,850	409,000	623,900	706,400
Number of children 6–23 months ever enrolled to receive MNPs	11,023	8171	6225	4295
Male	5588	4100	3109	2106
Female	5435	4071	3116	2189
Primary data participants and audio recordings				
Ghana Health Service staff interviewed^b^	5	5	5	5
Key informant interviews (KIIs) completed^c^	12	12	12	12
Focus group discussions (FGDs) completed^c^	6	6	6	6
Participants of discussion workshop^d^	10	10	10	10
Hours of key KII audio recordings	9.0	8.9	9.2	10.1
Hours of FGD audio recordings	7.5	7.8	7.8	8.5
Hours of discussion workshop audio recordings	2.3	1.7	1.4	2.5

^a^Data were from available records obtained from District Nutrition Officer

^b^Staff overseeing the program implementation, including the District Nutrition Officer and nurses from the major health facilities distributing the micronutrient powder (MNP)

^c^Mothers and caregivers who had previous or current direct experience with feeding children with MNP; they were selected if they were believed to have considerable knowledge about the Micronutrients Powder Initiative (MPI) and the attitudes of women in the communities towards MNP acceptability and consumption

^d^One staff member from each of 10 health facilities implementing the MPI

### Main experiences of mothers and caretakers

In general, caregivers reported that they accept the MNP for their children because they believe this supplement has health benefits. The caregivers reported having observed many positive impacts of MNP consumption in their children, including: weight gain; lower frequency of illnesses (e.g. diarrhea); increased appetite (in a positive sense), strength and energy; more regular bowel movements; and being able to walk independently relatively early compared with other siblings. A few of the quotes from caregivers as well as from a health worker are:

*“My child’s weight was always low every time I took her for weighing, but after I gave her some of the MNP, her weight is always increasing”* (FGD5, Adamu, Tain District).

*“My child initially did not like eating but after receiving and administering the MNP to him, it has boosted his appetite to eat.”* (KII7, Yagzore, Talensi District).

*“Some [mothers] testified and my wife also testified that it helps the children to walk sooner than usual” (Male, Ghana Health Service Staff, Tain District).*

Some caregivers were, however, reportedly skeptical about the MNPs being effective, particularly when the supplement was first introduced, while others reportedly refused to feed the supplements to their children or dropped out of the program because of what they think are side-effects of MNP consumption. This situation is shown by quotes from a key informant, a FGD panelist, and a GHS staff.

“*They think that the powder will not be effective so when they take the powder, they don’t take good care of it. They just use it anyhow, and some also say the child is not taking it but for some of us, our children are eating it”* (KII2, Debibi, Tain District).

*“When I added it to the koko [porridge], I realized my child had [frequent] stools. When I didn’t give it to the child the next day, I realized my child had passed the stool again, so I stopped giving it to my child because I thought it was the powder that was causing the stools. I complained to the nurse when I came for weighing. I have observed that my child doesn’t like it. It is not good for my child. My child passes a lot of stool so I have stopped giving it to him/her”* (FGD2, Kpedze, Ho West District).

*“When we introduced the MNPs, some mothers reported that their children were experiencing diarrhea. When they give it to the children, the children vomit. These were some of the challenges the mothers were facing. Due to these challenges, some mothers would collect the MNP but they wouldn’t give it as expected. They collect the MNP and dump it somewhere”* (Female, Discussion Workshop, Talensi District).

In at least one district (Tain), some of the advice received by caregivers concerning MNP administration to children appeared conflicting or inappropriate. One such advice prohibiting the mixing of MNP with “light porridge”, and another for caregivers whose children do not like eating food mixed with MNP were observed in the following quotes:

*“The mothers complain that if they don’t put the MNP into porridge, their children do not like to consume it. But they are not supposed to put it into light porridge”* (Male, Ghana Health Service Staff, Tain District).

*“I was told to mix the powder with my food and eat it hurriedly whilst my child suckled. I was told to take it so that the child would get the benefits of the supplement from sucking the breastmilk”* (FGD3, Bruhani, Tain District).

### Main experiences of the GHS program implementers

#### Increased patronage of CWC services

Frontline nurses reported that the introduction of the MPI has contributed to an increased CWC patronage by mothers and caregivers. That is, being enrolled in the program and the prospect of receiving MNPs upon attending the CWC have motivated many mothers to bring their children to CWCs each month. Some of the nurses explained as follows:

“*It increased the CWC attendance because the mothers know that the nurses will give them something when they attend the CWC*” (Male, Discussion Workshop, Tain District).

“*Initially, after completion of immunization, the children were no longer brought to the CWC. But now, because of the MPI program, they come to the CWC until the children are 24 months old*” (Female, Ghana Health Service Staff, Tolon District).

*“The mothers know that it is good to bring their children to the CWC always, because that is how they receive the MNP. They know that if they miss the CWC, they won’t get the MNP if the nurses do not visit them in the homes”* (Male, Ghana Health Service Staff, Ho West district).

#### Interest and appreciation among caregivers for the MPI targeting children

Health workers reportedly find that caregivers generally appreciate the MPI intervention targeting the children. This positive attitude of caregivers towards the MPI tends to encourage acceptability of the MNP thereby promoting program implementation. One of the health workers explained:

*“Mothers appreciate and like the intervention targeting the children”* (Male, Ghana Health Service Staff, Tolon District).

#### Certain organoleptic characteristics, side-effects, and MNP misuse negatively affecting program uptake

The reported complaints of various undesirable organoleptic characteristics of the MNP (e.g. it is reported to impart taste and colour to food) as well as possible side-effects of its consumption in some children appear to negatively affect program uptake. In addition, some mothers reportedly give the MNP to their children who are < 6 months of age, contrary to advice. Such children tend to experience more of the negative side-effects, thereby creating a bad impression about MNP consumption and reducing acceptability even among mothers of children ≥6 months of age. The following quotes explain:

“*When the baby passes stool, it smells, and there are indications of a disease. It occurred after giving the MNPs”* (FGD2, Bruhani, Tain District).

*“Some mothers say that when they mix the MNP with food, their children reject it, or they vomit, suffer diarrhea, or their stools turn black”* (Male, Ghana Health Service Staff, Ho West District).

“*Some mothers collect the MNP and give them to children who are not yet 6 months of age, only to conclude that the MNP is not good. Such mothers refuse to give the MNP to the children even when the children reach 6 months of age.*” (Male, Discussion Workshop, Tain District).

#### Challenges related to inadequate community sensitization

In at least two districts (Tain and Tolon), there were reported negative rumours that the MNP might be a birth control substance for children, and that, the MNP was being distributed for free because it is inferior. Some fathers reportedly prevent their children from taking the MNP because the fathers were not adequately engaged or sensitized about the program before the program was rolled out. Some participants explained:

*“When the implementation of the MPI started, some community members had a misconception that the MNP is a form of family planning. That is, we [health workers] don’t want their children to give birth to more children when they grow up. As a result, some men told their wives not to accept the MNP”* (Female, Discussion Workshop, Tolon District).

*“When you come to our communities, majority of girls 16 years and above have given birth, so they thought the MNP would cause the young children not to give birth that early when they grow up”* (Male, Discussion Workshop, Tain District).

*“Another challenge is that some fathers prevent their children from taking the MNP. Those fathers feel they are the head of the family, so no one can challenge their words or rules. Thus, although some women would collect the MNP from the nurses, the fathers do not allow them to feed the MNP to the children”* (Male, Discussion Workshop, Tain District).

#### Logistical or administrative challenges

Program implementers mentioned several logistical and administrative challenges, including: frequent MNP shortages in some districts (eg. Tain, Tolon and Talensi); challenges of using the current MNP distribution register which consists of loose sheets (as opposed to a bound booklet) and can therefore get lost or torn easily; poor means of transporting MNP from the regional stores to health facilities; lack of MNP counselling and information materials for caregivers; and inadequate training on MNP for program implementers. Some of the quotes from participants are:

“*One problem has to do with the register. The register consists of loose A3 sheets not bound together in a booklet form. So, it gets torn easily, especially when you carry it around for a while”* (Male, Discussion Workshop, Tain District).

*“The only material we have is the MPI training manual. We need posters we could paste at the health facilities. When we are counselling or educating the mothers, we have to remove a sachet from the box and use it to demonstrate to the mothers. I think that when mothers see the posters, they can understand it when we explain it to them. We didn’t get enough materials”* (Male, Ghana Health Service Staff, Talensi District).

*“The number of staff that was trained didn’t go well. With regard to the staff we trained, some of them have been transferred and others are in school. This is why we need refresher training.”* (Female, Ghana Health Service Staff, Tolon District).

### Successful strategies used to by program implementers

Frontline nurses found several successful strategies to address some of the challenges they experience in the implementation of the MPI:

#### One-on-one education and counselling sessions for caregivers

In many cases, frontline staff use “one-on-one” home contacts to educate caregivers about MNP and its potential benefits and side-effects. In addition, the nurses encourage caregivers to give the MNP to their children, since the potential benefits of the MNP outweigh the side-effects. They also advise mothers that: (a) the side effects are not uncommon to MNP consumption and that, they disappear, or they are easily treated and (b) children who do not initially like the MNP taste eventually get used to it.

*“I educate them about the need to encourage their children to consume the MNPs”* (Male, Ghana Health Service Staff, Ho West District).

*“In terms of bitterness, we tell them that as time goes on, the children will become used to it”* (Male, Ghana Health Service Staff, Tain District).

In addition, frontline staff try to involve other stakeholders such as community health volunteers, community health committee, mother-to-mother support groups, and father-to-father support groups to help educate community members on the side-effects of MNP and to dispel suspicions and rumours about the MPI.

*“We had to move quickly. We have mother-to-mother support groups, father-to-father support groups, and health committees. Through them we were able to manage the misconception that MNP is a form of family planning”* (Male, Discussion Workshop, Tain District).

*“With the involvement of the community health nurses (CHNs) and the volunteers, it was communicated very well to the community members for them to really understand the MNPs, its benefits, and side-effects” (*Female, Discussion Workshop, Tolon District).

#### Educating other health workers and community health volunteers not directly involved in the MPI implementation

Frontline staff often educate community volunteers and other health workers who are not directly involved in the MPI implementation. Once trained, these individuals also help in promoting the program. A participant explained as follows:

*“When we started the program, we, the community health nurses, were a lot so the other nurses were not part of it. Eventually, we also made sure they were aware of the program. Because there are some mothers who would go and show the MNP to the other nurses. So we wanted them to be aware so that whenever need be, they also encourage the mothers to feed the MNPs to the children. Also, the Community-Based Surveillance (CBS) volunteers in the communities are very influential. So, we also made sure they were informed about the program.”* (Male, Discussion Workshop, Tain District).

#### Asking mothers to share success stories

Frontline nurses often provide opportunities for caregivers who have fed MNP to their children and believe the children have benefited from the consumption (e.g. look stronger and more energetic than before) to share their success stories with others. This sharing of success stories helps to motivate other caregivers to feed the MNP to their children as well.

*“I know some mothers who are taking the MPI seriously, so I sometimes schedule those mothers to come to some of the CWC sessions. Then I use their children as examples so that others would see the difference. I don’t take mothers who are better off in the community. I take those who are similar to the majority of the mothers. Those mothers will now educate other mothers about the MNP”* (Male, Ghana Health Service Staff, Talensi District).

#### Tracing mothers and caregivers to their homes to address their concerns

As part of their routine home visits, frontline nurses and community health workers often trace mothers and caregivers to their homes to address the mothers’ concerns regarding the MNP consumption. The nurses also carry out home visits to monitor adherence to the advice about the use of the MNPs. One of the nurses explained as follows:

*“We make sure that those mothers who default, we trace to their home”* (Male, Discussion Workshop, Tain District).

#### Assuring mothers/caregivers that MNPs have been used elsewhere in Ghana before

To assuage mothers’ fears about the use of the MNPs, frontline nurses say they usually assure caregivers that MNP has been used elsewhere in Ghana before (i.e. in several previous research trials) and therefore they are not new in the country. This helps mothers and caregivers to commit to using the MNPs in the diets of their children. One KII participant explained:

*“The nurses explain to the mothers that the MNP come from the district health directorate, and that they have been used elsewhere in Ghana before being brought here”* (KII4, Debibi, Tain District).

### Facilitators and barriers to MPI implementation

Table 2 documents the facilitators and barriers to the MPI implementation and program uptake in the 4 districts focusing on 4 aspects of the program namely: (i) MNP acquisition at the district level and in the health facilities, (2) MNP distribution to mothers and caregivers, (3) records keeping and monitoring, and (4) MNP acceptability and consumption. The main facilitators of program implementation identified include: a generally regular MNP availability in health facilities, especially those in the Ho West district; use of several routine health contact points to introduce or distribute MNPs to mothers and caretakers; a fairly strong social mobilization strategy in which stakeholders including community health committee members, community volunteers, opinion leaders, chiefs, and queen mothers are engaged; good integration of the MPI with IYCF services during CWCs; strong involvement of the District Nutrition Officers providing support to frontline community health nurses; “one-on-one” education and counseling for mothers who report side effects; and tracing mothers/caregivers to their homes to monitor adherence and address concerns.

**Table 2:** Facilitators and barriers to Micronutrient Powder Initiative (MPI) implementation in 4 districts in Ghana based on data collected during a 2-month period from November to December 2019

Aspect of program	Facilitators^a^	Barriers^b^
*MNP acquisition at district level and in health facilities*	• Fairly regular availability of the MNPs at the health facilities ensure regular supply to mothers/caregivers	• Difficulty with transportation to collect MNP from regional or district capital to health facilities.
*MNP distribution to mothers and caregivers*	• Several contact points to introduce or supply MNP to mothers and caregivers, including: Child Welfare Clinics, community durbars, home visits by community health nurses, information centers, out-patient departments of health centers, radio, and outreaches. • Frontline nurses’ ability to deliver messages on MNP use to mothers/caregivers, including how to mix the MNP with home-prepared food. • Generally good social mobilization including involvement of community health committee members, community volunteers, opinion leaders, chiefs, and queen mothers to endorse and support the program. • Effective integration of the MNP program with the promotion of infant and young child feeding counselling reinforces each other. • Strong involvement of District Nutrition Officer offers strong support to frontline nurses. • Tracing mothers to their homes to monitor adherence and address the concerns.	• Absence of counselling cards, flip charts, posters, and leaflets specially made for the MNP distribution is making education about MNP difficult. • Inadequate training for the MNP Program managers and frontline nurses, and no refresher training thereafter. • High turn-over of health workers means that those initially trained on MNP may no longer be available to support implementation.
*Records keeping and monitoring*	• None identified	• Register used to record the particulars of beneficiary children are loose sheets with no serial numbers; it is difficult to handle these loose sheets, especially during outreach services, since the papers can easily get torn or lost. • Inadequate supply of registers to record the particulars of beneficiary children.
*MNP acceptability and consumption*	• Mothers’/caregivers’ and health workers’ motivation to improve or promote the health and nutrition of children in the district. • Understanding (among frontline nurses and women/caregivers) of the positive outcomes associated with MNPs consumption is necessary to motivate families to use MNPs regularly and appropriately. • Good counselling technique by frontline nurses, e.g. in Ho West, frontline nurses use the acronym “AFATVRAH” (Age, Frequency, Amount of food, Texture, Variety, Responsive feeding and Hygiene) to remind themselves regarding what they are supposed to counsel women/caregivers about. • Frontline nurses’ reported show of respect and patience as they deal with mothers and caregivers promote uptake of the program. • “One-on-one” education and counselling sessions for some mothers help to address specific issues such as potential side-effects, reported refusal to consume food mixed with MNP, misconception that MNPs are a birth control substance for children, etc. • Frontline nurses often provide the opportunity for mothers to share success stories about MNP consumption with others. • Food preparation demonstration to let mothers know that MNPs do not change taste of food considerably.	• Perceived inadequate community sensitization *before* program implementation • Lack of full community support for the program. • Skeptism of some mothers/caregivers about the MNPs being effective, especially during the early phase of the program. • Claims that the MNP changes the colour and/or taste of food (eg if mixed with a small quantity of food) tend to reduce acceptance. • Children’s experience of side-effects (whether perceived or actual) may reduce adherence to supplement intake. • Conflicting or inappropriate advice concerning MNP administration received by mothers/ caregivers. • Misconception and rumours that MNPs are a birth control substance for children tend to reduce acceptance.

^a^ Facilitators were defined as factors promoting the implementers’ ability and willingness to execute the program successfully and caregivers’ ability accept and utilize the program as promoted

^b^ Barriers were defined as factors militating against successful program implementation or uptake

The main barriers to program implementation identified are: several administrative and logistical challenges negatively affecting effective program delivery (eg. lack of counselling and communication materials specially designed for the MPI, challenges with the MNP distribution register, etc); negative rumours and suspicions towards the program (especially in the Tain and Tolon districts) causing some fathers to refuse their children’s enrolment into the program; absence of refresher training on the MPI for frontline health workers; children’s experience of side-effects causing some mothers not to use the MNPs; and undesirable organoleptic characteristics of the MNPs presumably enhanced by confusion about which foods to mix the supplements with and how.

## Discussion

The purpose of this study was to document the lessons learned from the implementation of the ongoing pilot MPI implemented in the Tain, Tolon, Talensi, and Ho West districts since 2017. Findings from the interviews and discussions with GHS staff involved in the program implementation, and from KIIs and FGDs with caregivers who have direct experience with the program suggest that caregivers generally accept the use of MNP for home-fortification of foods for their children. Caregivers said they have observed many positive effects of MNP consumption in their children, notably: increased appetite; less frequent illnesses; increased energy or strength; increased weight; and being able to walk independently relatively early.

Studies from similar MNP intervention programs in Ethiopia [18], Mali [19], and Peru [20] have reported positive impacts including improvements or increases in children’s weight, appetite, and energy/activity levels. While relatively few facilitators and barriers were identified for MNP acquisition at district level and in health facilities, and for MNP record keeping and monitoring, several were documented for MNP distribution to mothers and caregivers, and for MNP acceptability and consumption. Across various aspects of the MPI examined, relatively more facilitators were identified compared with the number of barriers, which suggests that the potential of the program to deliver micronutrients to children in the 4 districts may be promising.

The marked differences among the districts in terms of the amounts of MNP received and the number of children enrolled warrant discussion. First, the amounts of MNP received by the districts reflected the estimated numbers of children 6–23 months of age in the districts, which were used by UNICEF and GHS as the basis for determining the number of sachets required by each district, assuming that children 6–23 months constitute 6% of each district’s population. It is also possible that Ho West received the largest amount of MNP because it is closest to Accra and presumably would have relatively less difficulty collecting MNP from the national collection point in Accra. Besides their relatively small population sizes, the longer distances of Tolon and Talensi from Accra might contribute to why those districts received lower amounts of MNPs compared with Ho West or Tain. On the other hand, inadequate community sensitization and social mobilization on MNP use might account for the relatively low level of enrolment of children in the Ho West district compared with the other districts.

Given that the exact frequency of MNP distribution (eg. all 90 sachets at once every 6 months or 30 sachets every other month, etc.) is influenced by local contexts [28], it is unclear if the “3 months on, 3 month off” distribution regimen adopted by the GHS affected MNP acceptability, coverage and/or intake adherence. It is noteworthy that in this Ghana situation, the MNP distribution is integrated with monthly growth monitoring or CWC attendance. This allows relatively more frequent contacts with mothers and caregivers enrolled into the program, thereby increasing the potential of mothers and caregivers to understand and accept to use the MNPs [28].

The caregivers’ descriptions of their children’s “response” to MNP consumption are noteworthy. First, these descriptions appear to match what one would expect in micronutrient-deficient children responding positively to a micronutrient supplementation. For example, previous studies in Ghana [14, 30] showed that a greater percentage of children 6 months of age consuming multiple micronutrients were able to walk independently by 12 months of age than children receiving no intervention. Second, it is unknown from this qualitative cross-sectional study what proportion of mothers and caregivers feeding MNP to their children have made these observations, but these narratives are consistent with reports of persistently high prevalence of anemia among infants and children in Ghana [3].

There are several lessons learned or implications to be derived from these findings. First, the large number of children enrolled into the program, coupled with caregivers’ observation of positive response in the children suggests that incorporating the use of MNP into routine IYCF services at the CWCs is feasible within GHS and acceptable to mothers, and may help reduce anemia and possibly other micronutrient deficiencies among children 6–23 months of age in Ghana, if this intervention is carried out on a larger scale.

Another lesson learned is that a stronger community sensitization and social mobilization strategy would be necessary for the current pilot program as well as for any further scale-up. It sounds to reason that a weak community sensitization before the beginning of the pilot MPI might be a possible reason for the negative rumour and suspicion about the program in the Tain district, and some fathers’ refusal to allow their children to consume the MNP in some districts.

The reported complaints that children consuming MNP experience various side effects (e.g. diarrhea, darkening of stools, unusually smelly stools, and constipation) are probably not surprising, given that similar complaints have been reported from elsewhere in Sub-Saharan Africa, including change in the color, taste, smell and texture of food when mixed with MNP [18, 19, 31]. Similarly, mothers in Indonesia complained about MNP changing the taste and color of food [32, 33]. It is possible there was a bias of over-reporting these possible side effects, as observed in South Africa [34], with mothers and caregivers wrongly attributing any symptom to the addition of MNPs to the children’s food. In Uganda [35], mixing MNP with food prepared using soda ash (sodium carbonate) reportedly resulted in organoleptic changes to the food, but we are not aware that such cooking practice is common in the 4 districts. Here, a lesson learned is that moving forward, it would be important for frontline nurses to be able to advise caregivers adequately that such side-effects are not uncommon with MNP consumption, and therefore, they should not alarm caregivers excessively. This will require frontline nurses to be given refresher training from time to time, along with the necessary counselling and communication materials. Intensifying education for mothers and caregivers would improve the acceptability of the MNP, and help manage potential side-effects.

Another lesson learned is that several administrative and logistical challenges (e.g. frequent shortages of MNPs in some health facilities, lack of adequate means to transport MNPs from the regional or district level to the health facilities, etc.) remain, or do emerge from time to time. Previous studies on MNP have identified shortage of MNPs, lack of educational tools, and inadequate training as barriers to MPI implementation [20, 21, 36]. These challenges need to be consciously identified and managed, in order for the program to run effectively and thereby achieve its objectives.

A major strength of this study was the ability to interview and/or have discussions with both caregivers and program implementers from across several sub-districts within each district. Using various data collection strategies, including interviews with program managers, KIIs, FGDs and discussion workshops, was another strength. Additionally, since all the KIIs and FGDs were conducted in the local languages, and all interactions with study participants were audio-recorded before being transcribed verbatim, there was probably nothing that was said by any participant which was lost in the analysis. A few weaknesses deserve mention: first, the participants were not selected randomly and therefore it is possible that their responses may be biased in favour of the MPI; second, the number of mothers and caregivers involved in the KIIs and FGDs may be small compared with the total number whose children have been enrolled in the MPI; third, mothers and caregivers wanted the MPI to continue in their respective districts so it is possible that they gave socially desirable responses.

## Conclusions

The pilot MPI is well integrated into the GHS’ institutional routine, and that, MNP is generally well-accepted by mothers and caregivers. Suggested actions to help address existing challenges are to: strengthen social mobilization and community sensitization; intensify education for caregivers on regular and appropriate use of the MNP; equip frontline nurses with the skills and communication materials to manage side-effects; and provide the needed logistics for the program to run smoothly. Further research is needed to evaluate the effectiveness of the MPI in reducing anemia and micronutrient deficiencies among children 6–23 months of age in Ghana.

## Supplementary information

**Additional file 1: Supplementary file 1 Appendix 1**. Questionnaire guide for Staff of Ghana Health Service. This questionnaire guide was used to elicit information about program implementation and staff observations, experiences and lessons learned.

**Additional file 2: Supplementary file 2 Appendix 2**. Key Informants Interview Guide. This interview guide was used to collect information on mothers’ and caregivers’ observations or experiences and difficulties participating in the Micronutrient Powder Initiative.

**Additional file 3: Supplementary file 3 Appendix 3**. Focus Group Discussion (FGD) Guide. This discussion guide was used to collect information on mothers’ and caregivers’ observations or experiences participating in the Micronutrient Powder Initiative.

## Data Availability

The data sets used or analyzed during this current study are available from the corresponding author at reasonable request, and with permission from UNICEF. Dataset requests may be sent to Seth Adu-Afarwuah (ct3665@gmail.com).

## Abbreviations

CWC: Child Welfare Clinic
FGD: Focus Group Discussion
GHS: Ghana Health Service
GMP: Growth Monitoring and Promotion
IYCF: Infant and young child feeding
KII: Key informant interview
MNP: Micronutrient powder
MPI: Micronutrient Powder Initiative

## References

[1] Ritchie H, Roser M (2019). Micronutrient Deficiency.

[2] UG/GroundWork/UWisconsin-Madison/KEMRI/UNICEF (2017). Ghana Micronutrient Survey.

[3] GSS/GHS/ICF (2015). Ghana Demographic and Health Survey 2014.

[4] Bailey RL, West KP, Black RE (2015). The epidemiology of global micronutrient deficiencies. Ann Nutr Metab.

[5] Armar-Klemesu M, Osei-Menya S, Zakariah-Akoto S, Tumilowicz A, Lee J, Hotz C (2018). Using ethnography to identify barriers and facilitators to optimal infant and young child feeding in rural Ghana: implications for programs. Food Nutr Bull.

[6] WHO (2016). WHO guideline: use of multiple micronutrient powders for point-of-use fortification of foods consumed by infants and young children aged 6–23 months and children aged 2–12 years. WHO guideline: use of multiple micronutrient powders for point-of-use fortification of foods consumed by infants and young children aged 6–23 months and children aged 2–12 years.

[7] WHO: WHO guideline: Use of multiple micronutrient powders for point-of-use fortification of foods consumed by infants and young children aged 6–23 months and children aged 2–12 years. Licence: CC BY-NC-SA 3.0 IGO. World Health Organization, Geneva. 2016. Available from: https://apps.who.int/iris/bitstream/handle/10665/252540/9789241549943-eng.pdf?ua=1. [cited 2020 Feb 27]. 28079999

[8] UN SDG Knowledge Platform (2015). Sustainable Development Goals.

[9] WHO (2012). Comprehensive implementation plan on maternal, infant and young child nutrition. Sixty-fifth World Health Assembly, Geneva, 21–26 May 2012. Resolutions and decisions, annexes. WHA65/2012/REC/1.

[10] WHO (2015). The global strategy for women’s, children’s and adolescents’ health (2016–2023). Survive, thrive transform.

[11] Zlotkin S, Arthur P, Antwi KY, Yeung G (2001). Treatment of anemia with microencapsulated ferrous fumarate plus ascorbic acid supplied as sprinkles to complementary (weaning) foods. Am J Clin Nutr.

[12] Zlotkin S, Antwi KY, Schauer C, Yeung G (2003). Use of microencapsulated iron (II) fumarate sprinkles to prevent recurrence of anaemia in infants and young children at high risk. Bull World Health Organ.

[13] Christofides A, Asante KP, Schauer C, Sharieff W, Owusu-Agyei S, Zlotkin S (2006). Multi-micronutrient sprinkles including a low dose of iron provided as microencapsulated ferrous fumarate improves haematologic indices in anaemic children: a randomized clinical trial. Matern Child Nutr.

[14] Adu-Afarwuah S, Lartey A, Brown KH, Zlotkin S, Briend A, Dewey KG (2007). Randomized comparison of 3 types of micronutrient supplements for home fortification of complementary foods in Ghana: effects on growth and motor development. Am J Clin Nutr.

[15] Zlotkin S, Newton S, Aimone AM, Azindow I, Amenga-Etego S, Tchum K, Mahama E, Thorpe KE, Owusu-Agyei S (2013). Effect of iron fortification on malaria incidence in infants and young children in Ghana: a randomized trial. JAMA.

[16] Schauer C, Sunley N, Hubbell Melgarejo C, Nyhus Dhillon C, Roca C, Tapia G, Mathema P, Walton S, Situma R, Zlotkin S, et al. Experiences and lessons learned for planning and supply of micronutrient powders interventions. Matern Child Nutr. 2017;13(S1):e12494. 10.1111/mcn.12494 PMC5656916 28960875

[17] UNICEF (2017). NutriDash: Facts and figures – Nutrition Programme Data for the SDGs (2015–2030).

[18] Pelto GH, Tumilowicz A, Schnefke CH, Gebreyesus SH, Hrabar M, Gonzalez W, Wodajo HY, Neufeld LM (2019). Ethiopian mothers' experiences with micronutrient powders: perspectives from continuing and noncontinuing users. Matern Child Nutr.

[19] Roschnik N, Diarra H, Dicko Y, Diarra S, Stanley I, Moestue H, McClean J, Verhoef H, Clarke SE (2019). Adherence and acceptability of community-based distribution of micronutrient powders in southern Mali. Matern Child Nutr.

[20] Creed-Kanashiro H, Bartolini R, Abad M, Arevalo V (2016). Promoting multi-micronutrient powders (MNP) in Peru: acceptance by caregivers and role of health personnel. Matern Child Nutr.

[21] McLean J, Northrup-Lyons M, Reid RJ, Smith L, Ho K, Mucumbitsi A, Kayumba J, Omwega A, McDonald C, Schauer C (2019). From evidence to national scale: an implementation framework for micronutrient powders in Rwanda. Matern Child Nutr.

[22] Siekmans K, Bégin F, Situma R, Kupka R (2017). The potential role of micronutrient powders to improve complementary feeding practices. Matern Child Health.

[23] FAO/WHO (2004). Vitamin and mineral requirements in human nutrition.

[24] WHO (2018). Multiple Micronutrient Powders: For point-of-use fortification of foods consumed by Infants and children 6–23 months of age and children aged 2–12 years.

[25] MOH (1999). Ghana counseling cards for mothers and TBAs.

[26] MOH/World Vision (2015). Ghana National Community Health Worker Training Manual. Module 1: Community Health Basics Participant’s Manual.

[27] MOH/GHS (2009). Community Health Officer Training Manual (Facilitator's Guide) Volume 1.

[28] HF-TAG (2013). HF-TAG MNP Programmatic Guidance Brief: Programmatic guidance brief on the use of Micronutrient Powders for home fortification.

[29] Attride-Stirling J (2001). Thematic networks: an analytic tool for qualitative research. Qual Res.

[30] Prado EL, Adu-Afarwuah S, Lartey A, Ocansey M, Ashorn P, Vosti SA, Dewey KG (2016). Effects of pre- and post-natal lipid-based nutrient supplements on infant development in a randomized trial in Ghana. Early Hum Dev.

[31] D'Agostino A, Ssebiryo F, Murphy H, Cristello A, Nakiwala R, Otim K, Sarkar D, Ngalombi S, Schott W, Katuntu D (2019). Facility- and community-based delivery of micronutrient powders in Uganda: opening the black box of implementation using mixed methods. Matern Child Health.

[32] Inayati DA, Scherbaum V, Purwestri RC, Wirawan NN, Suryantan J, Hartono S, Bloem MA, Pangaribuan RV, Biesalski HK, Hoffmann V (2012). Combined intensive nutrition education and micronutrient powder supplementation improved nutritional status of mildly wasted children on Nias Island, Indonesia. Asia Pac J Clin Nutr.

[33] Sutrisna A, Vossenaar M, Poonawala A, Mallipu A, Izwardy D, Menon R, Tumilowicz A (2018). Improved information and educational messages on outer packaging of micronutrient powders distributed in Indonesia increase caregiver knowledge and adherence to recommended use. Nutrients.

[34] Smuts CM, Matsungo TM, Malan L, Kruger HS, Rothman M, Kvalsvig JD, Covic N, Joosten K, Osendarp SJM, Bruins MJ, et al. Effect of small-quantity lipid-based nutrient supplements on growth, psychomotor development, iron status, and morbidity among 6- to 12-mo-old infants in South Africa: a randomized controlled trial. Am J Clin Nutr. 2019;109(1):55–68. 10.1093/ajcn/nqy282 PMC6358035 30649163

[35] Ford ND, Ruth LJ, Ngalombi S, Lubowa A, Halati S, Ahimbisibwe M, Baingana R, Whitehead RD, Mapango C, Jefferds ME (2020). An integrated infant and young child feeding and micronutrient powder intervention does not affect Anemia, Iron status, or vitamin a status among children aged 12-23 months in eastern Uganda. J Nutr.

[36] Brewer JD, Santos MP, Román K, Riley-Powell AR, Oberhelman RA, Paz-Soldan VA (2020). Micronutrient powder use in Arequipa, Peru: barriers and enablers across multiple levels. Matern Child Nutr.

