# Supplementary material for: Lessons learned from implementing the pilot Micronutrient Powder Initiative in four districts in Ghana
Source: BMC Nutr. 2020 Nov 9;6:50. doi: 10.1186/s40795-020-00382-3 (PMC7650146; doi:10.1186/s40795-020-00382-3)
Supplement: Supplementary file 1 — Additional file 1: Supplementary file 1 Appendix 1. Questionnaire guide for Staff of Ghana Health Service. This questionnaire guide was used to elicit information about program implementation and staff observations, experiences and lessons learned. [file 40795_2020_382_MOESM1_ESM.docx]

**Appendix 1: Questionnaire guide for Staff of Ghana Health Service**

1. How does the hospital/clinic obtain or acquire the multiple micronutrient powder (MNP) for this program?
2. How does the hospital/clinic deliver or distribute the MNPs to mothers and caregivers in this program?
3. What sequence of activities did/do you perform during the implementation of the project?
4. What key decisions did/do you take during the implementation of the project?
5. What are your main observations or experiences, as a staff of this hospital/clinic, participating in the implementation of this program?
6. What are your main observations or experiences with regards to the communities, mothers and caregivers taking part in the program and their attitudes toward the program?
7. What important problems or difficulties did/do you have to deal with at any time during the program implementation?
8. What innovative strategies were/have been put in place to address those problems or difficulties.
9. What went well as planned or intended?
10. What did not go well as planned or intended?
11. Did you observe/have you observed any unintended consequences as a result of the program implementation?
12. What is your overall assessment of the program in achieving its goals?
13. What lessons, if any, have you learned during the implementation this program?
14. What would be done differently, if the program was to be implemented all over again?
15. What recommendations or advice would you offer to others implementing a similar program on a pilot basis and on a larger scale?
