# Supplementary material for: Lessons learned from implementing the pilot Micronutrient Powder Initiative in four districts in Ghana
Source: BMC Nutr. 2020 Nov 9;6:50. doi: 10.1186/s40795-020-00382-3 (PMC7650146; doi:10.1186/s40795-020-00382-3)
Supplement: Supplementary file 2 — Additional file 2: Supplementary file 2 Appendix 2. Key Informants Interview Guide. This interview guide was used to collect information on mothers’ and caregivers’ observations or experiences and difficulties participating in the Micronutrient Powder Initiative. [file 40795_2020_382_MOESM2_ESM.docx]

**Appendix 2: Key Informants Interview Guide**

1. Please tell me about your influence in your community.
   1. Interviewer: Probe for influence related to nutrition and IYCF (EBF/CF/ Continued BF.
   2. What do you think community members see as your influence?
   3. Have you worked with or do you work and have any connections with NGOs, Ghana Health Service, CHIP Centers, community groups?
2. Tell us what you know about the micronutrient powder (MNP) given to mothers and caregivers to add to their children’s food.
3. How was the Micronutrient Powder Initiative (MPI) introduced to mothers and caregivers in this community? /How did mothers and caregivers get to know about the MPI program or how did they start receiving the micronutrient powder (MNP) for their children?
4. What did/do the mothers in the community think about the MNP supplement or the program?
5. What did/do the health facilities staff do whilst running the program? For example, what advice did/do they give to mothers regarding the use of the MNPs?

1. What actions or approaches taken by the health workers went well?
2. Which actions or approaches taken by the health workers did not go well?
3. What examples of unexpected results did/do mothers and caregivers encounter?
4. What are your main observations or experiences or those of mothers in this community regarding giving the MNP to children?
5. What important problems or difficulties did/do you or mothers/caregivers in this community have to deal with whilst feeding children with MNPs?
6. Were/are there any changes that you observed/have observed in children who are receiving or have received MNP?
7. What is your overall assessment or opinion about the program?
8. What lessons, if any, have you learned during the implementation this program?
9. What advice would you offer to other mothers/caregivers in this community regarding the use of MNPs now or in future?
