# Supplementary material for: Lessons learned from implementing the pilot Micronutrient Powder Initiative in four districts in Ghana
Source: BMC Nutr. 2020 Nov 9;6:50. doi: 10.1186/s40795-020-00382-3 (PMC7650146; doi:10.1186/s40795-020-00382-3)
Supplement: Supplementary file 3 — Additional file 3: Supplementary file 3 Appendix 3. Focus Group Discussion (FGD) Guide. This discussion guide was used to collect information on mothers’ and caregivers’ observations or experiences participating in the Micronutrient Powder Initiative. [file 40795_2020_382_MOESM3_ESM.docx]

**Appendix 3: Focus Group Discussion (FGD) Guide**

1. First, please let us all introduce ourselves by telling a bit about ourselves. For example, tell us about your children and family. [*Moderator: Go around and introduce. Notetaker should take detailed notes on how they describe themselves, without capturing names*].
2. Tell us what you know about the micronutrient powder (MNP) given to mothers and caregivers to add to their children’s food.
3. How was the Micronutrient Powder Initiative (MPI) introduced to mothers and caregivers in this community? /How did mothers and caregivers get to know about the MPI program or how did they start receiving the micronutrient powder (MNP) for their children?
4. What did/do the mothers/caregivers in the community think about the MNP supplement or the program?
5. What did/do the health facilities staff do whilst running the program? For example, what advice did/do they give to mothers/caregivers regarding the use of the MNPs?

1. What actions or approaches taken by the health workers went well?
2. Which actions or approaches taken by the health workers did not go well?
3. What examples of unexpected results did/do mothers and caregivers encounter?
4. What are your main observations or experiences or those of mothers in this community regarding giving the MNP to children?
5. What important problems or difficulties did/do you or mothers/caregivers in this community have to deal with whilst feeding children with MNPs?
6. Were/are there any changes that you observed/have observed in children who are receiving or have received MNP?
7. What are your overall assessments or opinions about the program?
8. What lessons, if any, have you learned during the implementation this program?
9. What advice would you offer to other mothers/caregivers in this community regarding the use of MNPs now or in future?
